# Giant diverticulum‐ A rare complication of a common surgical condition

**DOI:** 10.1002/ccr3.534

**Published:** 2016-04-13

**Authors:** Vanessa Cubas, Stephen T. Ward, Jan Dmitrewski

**Affiliations:** ^1^ Queen Elizabeth HospitalMindelsohn WayBirminghamB15 2THUK; ^2^ National Institute for Health Research (NIHR) Birmingham Liver Biomedical Research Unit (BRU)University of BirminghamVincent DriveBirminghamB15 2TTUK

**Keywords:** Diverticular disease, general surgery, giant diverticulum, sigmoid diverticulum

## Abstract

A gentleman presented with abdominal distension and pain. CT confirmed a 20 cm sigmoid diverticulum. A giant diverticulum, typified by diverticula greater than 4 cm, often requires colonic resection. Fewer than 200 cases have been reported, most measuring 7–15 cm. I present a rare complication of a common surgical condition with images.

## Introduction

## What is this condition?

### Giant colonic diverticulum

**Figure 1:**
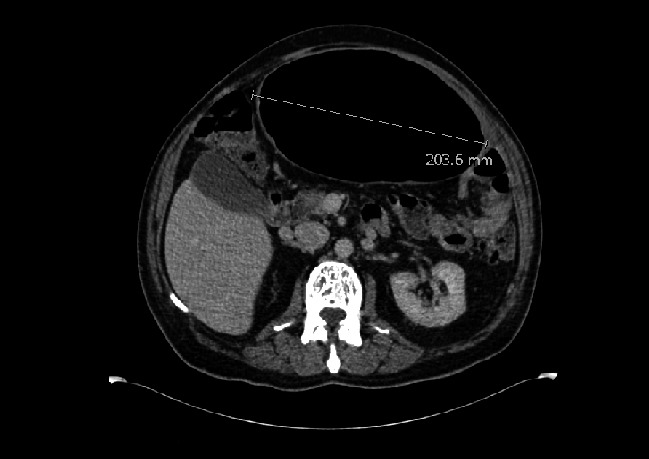
CT showing a 20.3 cm sigmoid mass in keeping with giant diverticulum.

**Figure 2:**
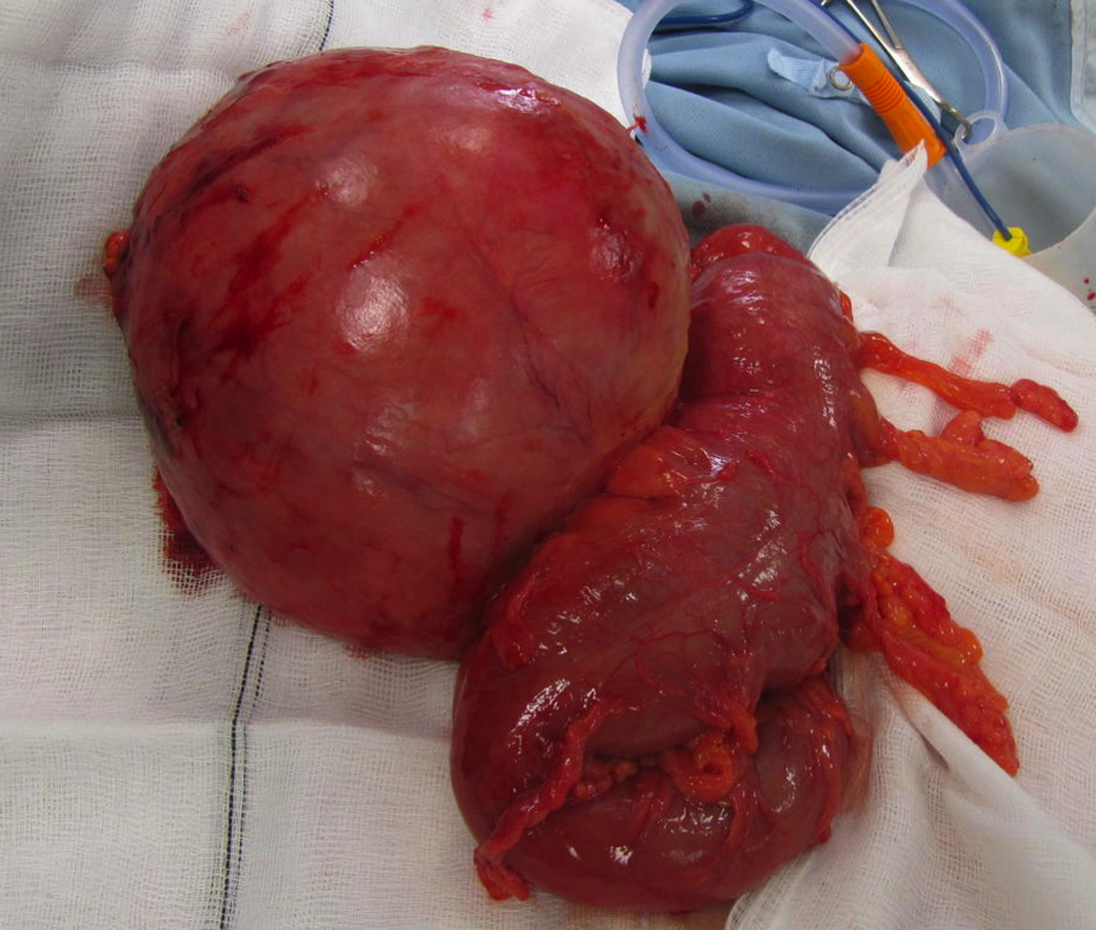
Specimen following sigmoidectomy.

A 65‐year‐old gentleman presented with abdominal distension and pain. CT confirmed a 20 cm sigmoid diverticulum (Fig. 1). Although he was clinically well a sigmoidectomy was performed due to the risk of perforation (Fig. 2). The patient was discharged 3 days later.

Giant diverticulum, typified by diverticula greater than 4 cm, is a rare manifestation of diverticular disease of the colon. It affects the sigmoid in 90% of cases 1. Since it was first described by Hughes and Green in 1953, there have been fewer than 200 cases reported with most measuring between 7 and 15 cm. Although etiology is not clearly understood, it is believed that a one‐way valve is created at the communication between the colon and the sac leading to air entrapment and a gradual distension of the sac 2. Treatment usually entails surgery: colonic resection with a primary anastomosis.

## Conflict of Interest

None declared.
